# Effect of pre-visit computer-assisted history-taking with a structured copy-ready summary on documentation completeness in screening for inflammatory rheumatic diseases: a randomized pilot study

**DOI:** 10.1186/s12913-026-15808-0

**Published:** 2026-10-09

**Authors:** Anna Roemer, Tim Wilhelmi, Daniel Fink, Manuel Göbel, Juliana Hack, Jonathan Bamberger, Felix Mühlensiepen, Karolina Gente, Sebastian Kuhn, Philipp Klemm, Johannes Knitza

**Affiliations:** 1 Institute for Digital Medicine, School of Medicine, Philipps-Universität Marburg, Baldingerstrasse 1, 35043 Marburg, Germany; 2 Department of Rheumatology and Clinical Immunology, Justus-Liebig University Giessen, Kerckhoff Campus, Bad Nauheim, Germany; 3 Rheumazentrum Mittelhessen, Bad Endbach, Germany; 4 Department of Dermatology and Allergology, School of Medicine, Philipps-Universität Marburg, Marburg, Germany; 5 School of Medicine, Center for Orthopaedics and Trauma Surgery, Philipps-Universität Marburg, Marburg, Germany; 6 Center for Health Services Research, Faculty for Health Sciences Brandenburg, Brandenburg Medical School Theodor Fontane, Ruedersdorf, Germany; 7 Internal Medicine V, Hematology, Oncology and Rheumatology, Heidelberg University Hospital, Heidelberg, Germany

**Keywords:** Medical history taking, Computer-assisted history-taking, Documentation completeness, Rheumatology, Screening, Digital health

## Abstract

**Background:**

Clinical encounters in outpatient care are constrained by high demand, limited consultation time, and documentation requirements. Computer-assisted history-taking (CAHT) may improve the structured collection of patient information before consultations and disease screening, but evidence is limited. This randomized pilot study evaluated whether pre-visit CAHT combined with a copy-ready summary increased documentation completeness and assessed patient and physician acceptance.

**Methods:**

MAESTRI was a multicenter, parallel-group, randomized pilot study conducted across rheumatology, dermatology, and orthopedics outpatient services. Adults at increased risk of an underlying inflammatory rheumatic disease (IRD) were randomized to pre-visit CAHT or usual care. Specialty-specific questionnaires generated structured narrative summaries covering predefined screening-relevant items. The primary outcome was documentation completeness, assessed by a blinded reviewer as the percentage of these predefined items present in the final clinical documentation. Secondary and exploratory outcomes included patient and physician acceptance, usability ratings, and free-text feedback.

**Results:**

Of 45 screened patients, 43 were randomized and analyzed. The mean age was 54.2 years (SD 14.3), and 29 participants (67.4%) were female. All intervention-group clinical notes contained the generated summary. Overall median documentation completeness was 100.0% (IQR 100.0-100.0) in the intervention group compared with 46.9% (IQR 10.0-68.8) in the control group (*p* < 0.001), with consistent differences across rheumatology, dermatology, and orthopedics. CAHT acceptance was high among both patients and physicians, with 41 of 43 patients (95.3%) willing to complete and the six responding physicians (100%) willing to offer digital pre-visit questionnaires before future appointments. Qualitative feedback was predominantly positive, with patients reporting that pre-visit questionnaires could improve consultation preparation, support recall of symptoms, and help physicians focus the visit on relevant issues. Some participants emphasized that digital questionnaires should complement, rather than replace, direct physician interaction.

**Conclusions:**

Pre-visit CAHT combined with a structured, copy-ready summary substantially increased the completeness with which predefined screening-relevant information was transferred into clinical documentation while also suggesting high acceptance among both patients and physicians. These findings support further evaluation in a larger trial assessing workflow impact and clinical impact.

**Trial registration:**

The study was registered at German Clinical Trials Register (DRKS; ID: DRKS00036519). Date of Registration: 2025-03-31.

**Supplementary Information:**

The online version contains supplementary material available at https://doi.org/10.1186/s12913-026-15808-0.

## Introduction

Clinical encounters are increasingly constrained by limited consultation time, high demand, and documentation requirements [1–3]. In specialty care, these pressures may reduce the time available for comprehensive history taking and may contribute to incomplete documentation, delayed recognition of relevant differential diagnoses and inefficient use of face-to-face consultation time.

Pre-visit computer-assisted history taking (CAHT) offers a practical approach to collect structured patient-reported information before the clinical encounter. Prior work suggests that automated history taking can capture more complete clinical information, reduce documentation time [4–8], support pre-visit preparation and allow patients to answer questions at their own pace [4, 6, 7]. In addition, structured pre-visit information may help ensure that clinically relevant symptoms, risk factors, and comorbidities are systematically captured and available during the consultation. This is particularly relevant for inflammatory rheumatic diseases, where timely recognition and referral are essential to enable early treatment and prevent irreversible damage [9, 10]. Structured history taking may be especially useful in at-risk populations, including patients with psoriasis or chronic back pain, in whom targeted screening can support earlier detection of psoriatic arthritis or axial spondyloarthritis [7–10]. For patients with psoriasis, regular screening for psoriatic arthritis every 6 to 12 months is recommended, but implementation in routine outpatient care can be challenging within time-constrained clinical workflows [7]. Similarly, for broader screening of inflammatory rheumatic joint diseases, international experts have developed the Inflammatory Arthritis Detection Tool using a Delphi consensus process [13]. The European Alliance of Associations for Rheumatology has also recommended considering digitally supported patient pre-assessment as part of strategies to improve rheumatology care [11].

Despite the potential benefits of CAHT and existing recommendations to screen at-risk patients for rheumatic diseases, implementation in routine care remains limited [7]. Moreover, evidence on the use of CAHT to support screening for inflammatory rheumatic diseases is scarce [12], particularly across different specialty settings. Randomized evidence on the effect of pre-visit CAHT on documentation completeness in this context remains limited.

To address these evidence and implementation gaps, the MAESTRI study evaluated pre-visit CAHT combined with a structured, copy-ready summary to support screening for inflammatory rheumatic diseases across rheumatology, dermatology, and orthopedics services. The primary objective was to determine whether CAHT improves the completeness of medical documentation. Secondary objectives were to explore patient and physician acceptance of pre-visit computerized history taking in routine clinical care.

## Methods

MAESTRI was a multicenter, parallel-group, randomized pilot study comparing rheumatology-specific pre-visit computerized history taking with usual pre-visit procedures. All participants provided written informed consent before study participation. The study was conducted in accordance with the ethical principles of the Declaration of Helsinki and the Ethics Committee of Philipps University Marburg approved the study on March 24 2025 (reference: 25–35 BO). The study was prospectively registered in the German Clinical Trials Register (DRKS; DRKS00036519). Reporting followed the Consolidated Standards of Reporting Trials (CONSORT) guideline for randomized trials (supplementary file).

### Population

Eligible participants were adults aged 18 years or older who were at increased risk of an underlying rheumatic disease and had a scheduled appointment in one of the participating clinical settings. These included newly referred patients at two rheumatology centers, one university-based and one non-university-based, as well as patients with psoriasis attending a university dermatology outpatient clinic and patients with back pain attending a university orthopedic outpatient clinic.

In addition, participants were required to have access to a personal email address and an internet-enabled device, such as a smartphone, tablet, or laptop, with a web browser to access the questionnaire via link or QR code. Sufficient German language proficiency was required to understand the study information and complete the digital questionnaires.

### Recruitment, consent and randomization

Patients were informed about the study by treating physicians, nurses, or administrative staff. All participants provided written informed consent before enrollment and randomization. No financial compensation was offered for participation. After consent had been obtained, participants were randomized between November 4 and November 27, 2025, in a 1:1 allocation ratio to the intervention or control group. Randomization was performed using a computer-generated random sequence and was conducted by an independent researcher. Enrolling investigators had no access to the allocation sequence. Participants were not blinded to group assignment.

### Intervention and control

Participants allocated to the intervention group received an invitation link before their scheduled appointment to complete rheumatology-specific questionnaires (supplementary file) using Idana software (Idana AG, Freiburg, Germany). Separate questionnaires were developed for each of the three clinical settings: rheumatology, dermatology/psoriasis, and orthopedics/back pain. The questionnaires were designed in collaboration with the respective heads of each clinical unit. The questionnaires covered predefined screening-relevant items that were subsequently used to assess documentation completeness.

The rheumatology questionnaire was based on previous questionnaires for newly referred patients [13–15] and was designed to provide standardized minimal screening for inflammatory rheumatic diseases and key comorbidities; this questionnaire comprised 42 items. For patients in the psoriasis clinic, the questionnaire focused on information relevant to screening for psoriatic arthritis. It was based on the Psoriasis Epidemiology Screening Tool (PEST) [16] and slightly extended, resulting in 10 items. For patients in the back pain clinic, the questionnaire focused on screening for axial spondyloarthritis. It included the five-parameter criteria developed by the Assessment of SpondyloArthritis international Society [17] and additional items from a previously identified [18] optimal screening tool for axial spondyloarthritis in patients with chronic back pain [19]; this questionnaire comprised 20 items.

In collaboration with a senior consultant of participating units, structured text templates were developed for each setting. These templates were automatically generated from the patient-completed questionnaires and were designed to be copied into the clinical documentation. Treating physicians of patients in the intervention group were given access to Idana and could use the generated summary templates during routine care. Patients and treating physicians in the intervention group were also asked to complete a short evaluation questionnaire. Participants and treating physicians in the control group received only a reduced evaluation questionnaire and otherwise followed routine care. Idana is commercially available under a practice-level, usage-based subscription rather than per-user licensing. According to the vendor’s publicly listed German pricing (https://idana.com/preise/, accessed September 14, 2026), packages started at EUR 79 per month plus value-added tax with annual payment. The study did not conduct a formal economic evaluation.

### Outcomes

The primary outcome was documentation completeness, defined as the proportion of predefined specialty-specific screening items present in the final clinical documentation. Documentation completeness was evaluated by a blinded reviewer using specialty-specific checklists based on the approach described by Wahlberg et al. [20]. The specialty-specific item scoring checklists are provided with control group results in the supplementary file. Because the number of items differed across specialties and scoring instruments, raw documentation scores were converted to percentages to enable comparison across groups and specialties. The checklists were study-specific scoring instruments and had not undergone external validation. All records were scored once by a single blinded reviewer; no inter-rater or intra-rater reliability assessment was performed.

Secondary outcomes were patient and treating physician acceptance to use computerized pre-visit history-taking in the future. These outcomes were assessed using 7-point Likert scales, with 1 indicating “strongly disagree,” 4 indicating “neutral,” and 7 indicating “strongly agree.”

Other outcomes included additional items rated on 7-point Likert scales and participants could provide free-text feedback on their general perception of computerized history-taking. Patients and physicians in both groups were asked about their general preference for digital rather than paper-based questionnaires and whether they believed that computerized history-taking would enable better use of physician-patient consultation time. Physicians were additionally asked whether they believed that computerized history-taking would increase their productivity. In the intervention group, patients and physicians completed four additional items adapted from the short version of the German Telehealth Usability Questionnaire [21]. These items assessed whether they would use computerized history-taking again in the future, whether the system was easy to use, whether the design was pleasant, and whether errors were easy to correct. Patients in the intervention group were additionally asked whether the time required to complete the questionnaire was reasonable and whether the questions were easy to understand.

### Statistical analysis

Analyses were descriptive and exploratory, consistent with the pilot-study design. No formal sample size calculation for efficacy testing was performed, as to the best of our knowledge, no sufficiently comparable prior studies were available to inform assumptions for an efficacy-based sample size calculation. Baseline characteristics are presented as mean and standard deviation for age and as counts and percentages for categorical variables. Likert-scale responses are summarized as absolute counts and percentages and were grouped as strongly disagree (1), disagree (2–3), neutral (4), agree (5–6), and strongly agree (7). Agree total was defined as scores 5–7. Documentation completeness percentages and ordinal Likert outcomes were summarized using medians and interquartile ranges. Original documentation scores were converted to percentages of the respective maximum attainable score before analysis. Missing values were not imputed; analyses were based on available valid responses for each outcome. Intervention-control comparisons were performed using two-sided Mann-Whitney U tests, without adjustment for multiplicity. The Mann-Whitney U tests used average ranks with tie correction and a normal approximation with continuity correction. Statistical significance was interpreted using alpha = 0.05. Free-text feedback was analyzed descriptively using an inductive thematic approach. Two researchers independently read and coded responses iteratively to identify recurring patterns. Initial codes were derived inductively from the data without reference to a predefined framework, then discussed and consolidated into overarching themes by consensus. Data handling and figure generation were performed using Python 3.12.13 with pandas 2.2.3, numpy 2.3.5, and openpyxl 3.1.5.

## Results

**Fig. 1:**
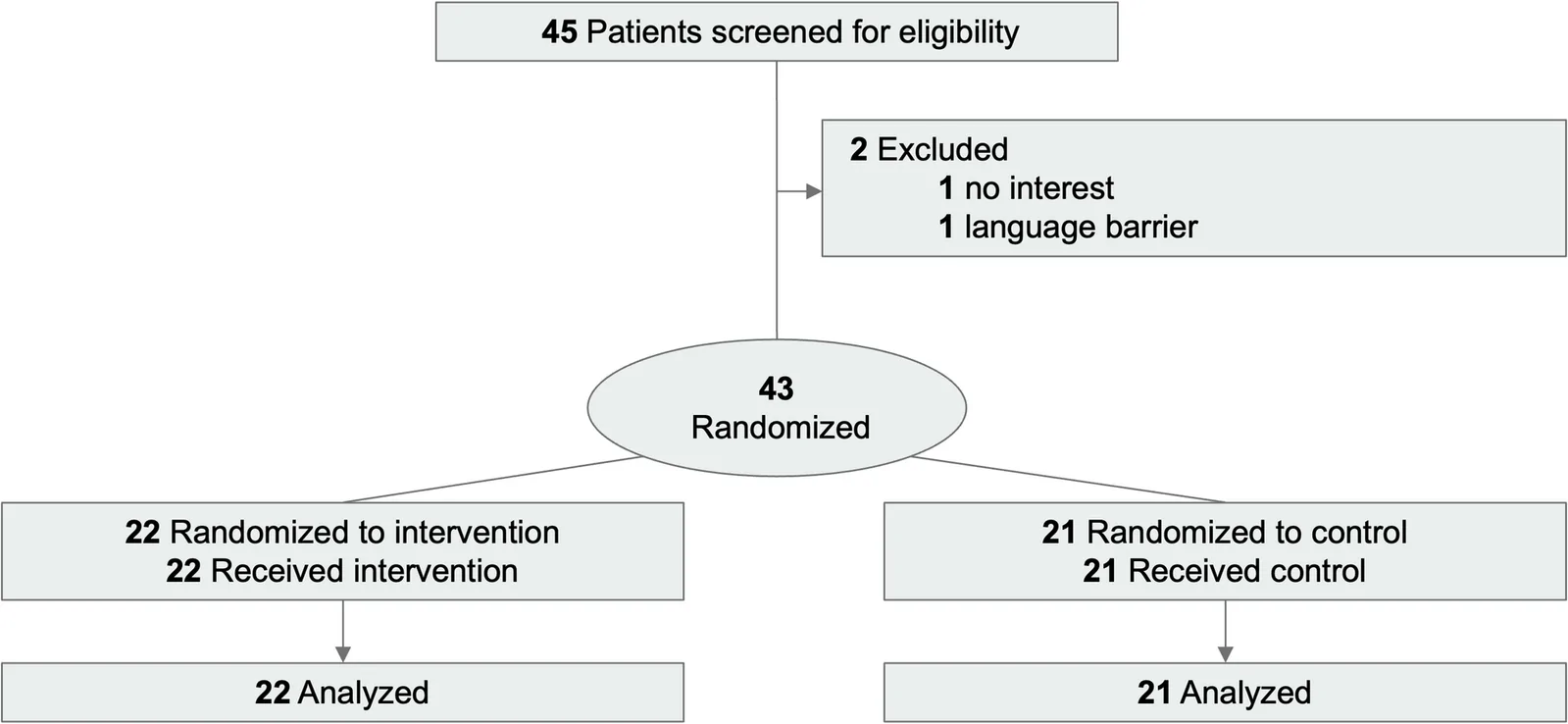
Trial participant flow diagram

Of 45 patients screened, 43 were randomized and included in the analyses (Fig. 1). Twenty-two participants were allocated to the intervention group and 21 to the control group. The analyzed cohort had a mean age of 54.2 years (SD 14.3), 29 participants (67.4%) were female, and the largest proportion was recruited from rheumatology clinics (41.9%; Table 1). The groups were similar in mean age and specialty distribution, although the intervention group included a higher proportion of women (77.3% vs. 57.1%) and the control group included a higher proportion of participants recruited from orthopedics (33.3% vs. 22.7%).

**Table 1:** Patient demographics

Characteristic	Total (*N* = 43)	Intervention group (*n* = 22)	Control group (*n* = 21)
Age, mean (SD), y	54.2 (14.3)	53.2 (12.7)	55.3 (16.0)
**Gender**,** n (%)**			
Female	29 (67.4)	17 (77.3)	12 (57.1)
Male	14 (32.6)	5 (22.7)	9 (42.9)
**Medical specialty**,** n (%)**			
Rheumatology	18 (41.9)	10 (45.5)	8 (38.1)
Dermatology	13 (30.2)	7 (31.8)	6 (28.6)
Orthopedics	12 (27.9)	5 (22.7)	7 (33.3)

### Primary outcome

**Fig. 2:**
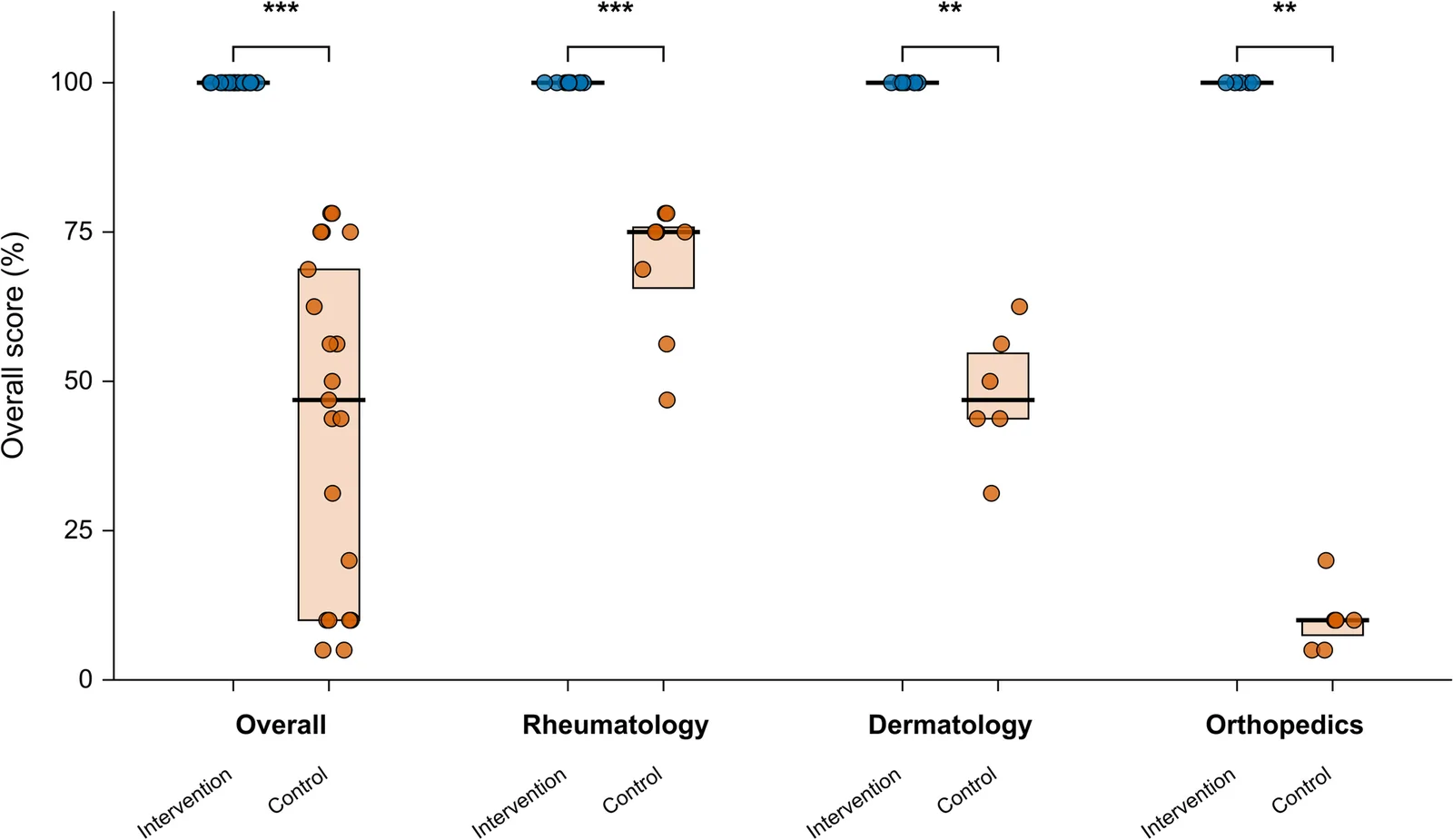
Documentation completeness in intervention and control groups

All 22 intervention-group clinical notes contained the CAHT-generated structured summary, which had been copied largely verbatim into the clinical documentation. Overall, the median documentation score was 100.0% (IQR 100.0–100.0) in the intervention group, compared with 46.9% (IQR 10.0–68.8) in the control group (*p* < 0.001), see Fig. 2; Table 2. This difference was observed across all specialty groups. Control group scores were 75.0% (IQR 65.6–75.8) in rheumatology, 46.9% (IQR 43.8–54.7) in dermatology, and 10.0% (IQR 7.5–10.0) in orthopedics, compared with 100.0% (IQR 100.0–100.0) in the intervention group for each specialty (rheumatology *p* < 0.001; dermatology *p* = 0.001; orthopedics *p* = 0.003). The exact results of missing and absent items for the control groups are displayed in the supplementary file.

Jitter plots show individual documentation completeness scores, expressed as percentages, for intervention and control participants overall and stratified by specialty. Scores were converted to percentages to enable comparison across specialties and scoring instruments. Box plots indicate the median and interquartile range. Statistical significance between groups is shown above each comparison (**p < 0.01; ***p < 0.001).

**Table 2:** Documentation completeness by randomized group

Group	Intervention group, *n*	Intervention median (IQR) %	Control group, *n*	Control median (IQR) %	*p* value
Overall	22	100.0 (100.0-100.0)	21	46.9 (10.0-68.8)	< 0.001
Rheumatology	10	100.0 (100.0-100.0)	8	75.0 (65.6–75.8)	< 0.001
Dermatology	7	100.0 (100.0-100.0)	6	46.9 (43.8–54.7)	0.001
Orthopedics	5	100.0 (100.0-100.0)	7	10.0 (7.5–10.0)	0.003

### Secondary outcome

Acceptance to complete digital pre-visit questionnaires was high in both randomized groups and did not differ significantly between intervention and control participants (Table 3). Overall, 41 of 43 (95.3%) patients agreed or strongly agreed that they would be willing to complete digital questionnaires before future physician visits. The median score was 7.0 (IQR 6.0–7.0) in both groups (*p* = 0.242). No statistically significant differences were observed within rheumatology, dermatology or orthopedics. Only six physician evaluations were available (2 rheumatologists, 4 orthopedics) and should therefore be interpreted cautiously. All six (100%) agreed or strongly agreed that they would be willing to offer digital pre-visit questionnaires before future appointments.

**Table 3:** Patient acceptance to use pre-visit computerized history-taking in the future by randomized group

Group	Intervention group, *n*	Intervention median (IQR), %	Control group, *n*	Control median (IQR), %	*p* value
Overall	22	7.0 (6.0–7.0)	21	7.0 (6.0–7.0)	0.242
Rheumatology	10	6.5 (6.0–7.0)	8	6.0 (5.0–7.0)	0.442
Dermatology	7	7.0 (7.0–7.0)	6	6.5 (6.0–7.0)	0.293
Orthopedics	5	7.0 (7.0–7.0)	7	7.0 (6.5-7.0)	0.748

### Other outcomes

**Fig. 3:**
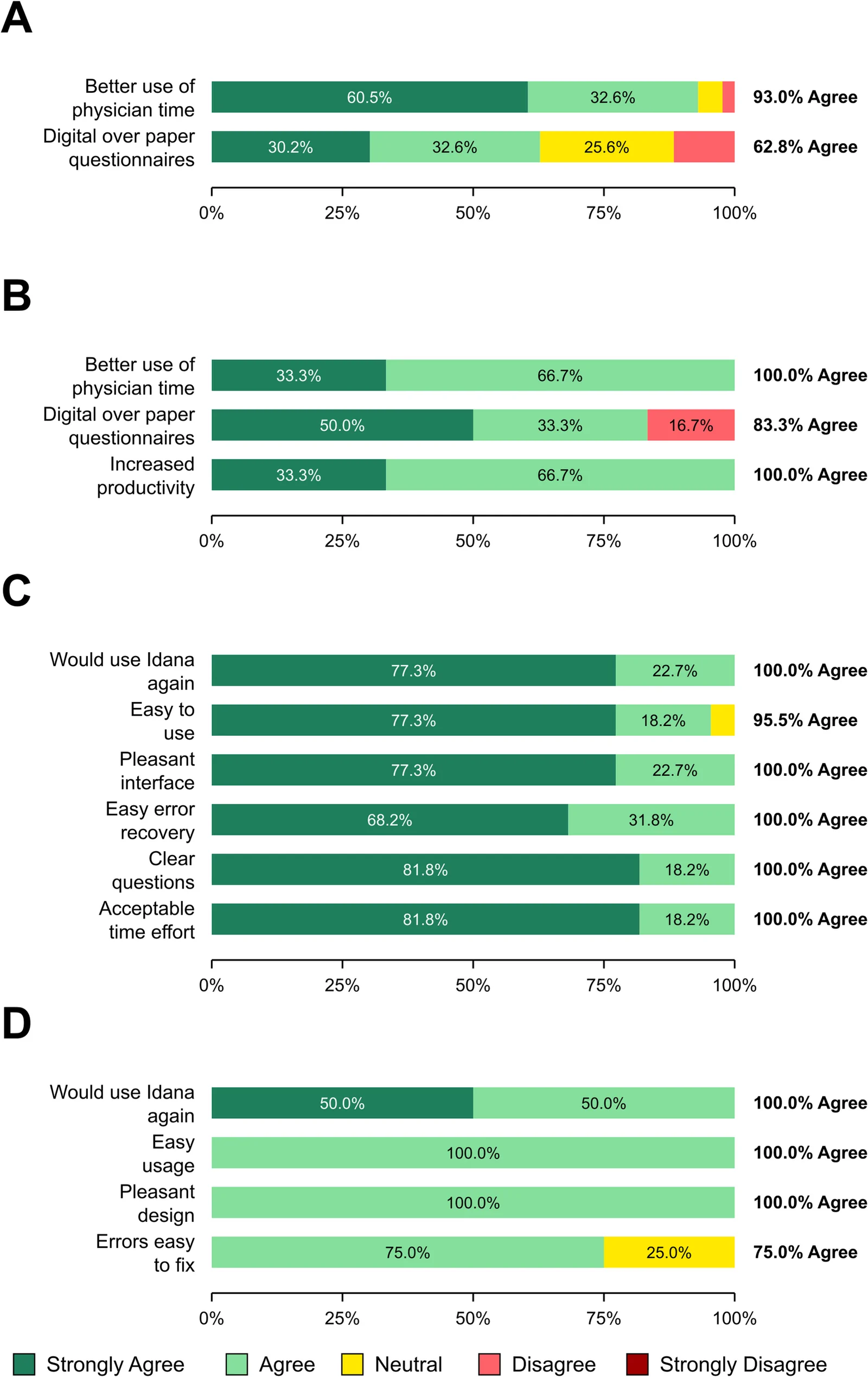
Patient and physician evaluation of digital pre-visit questionnaires. Stacked percentage bar charts show Likert-scale responses grouped as strongly disagree, disagree, neutral, agree, and strongly agree. Panel **A** shows overall patient evaluation items (*n* = 43), panel **B** shows overall physician evaluation items (*n* = 6), panel **C** shows patient usability ratings among intervention participants (*n* = 22), and panel **D** shows physician usability ratings among intervention physicians (*n* = 4). Percentages indicate the proportion of respondents in each response category; values on the right summarize the combined proportion agreeing or strongly agreeing

Overall, 40 of 43 participants (93.0%) agreed or strongly agreed that digital pre-visit questionnaires could help use physician time more meaningfully (Fig. 3). Twenty-seven participants (62.8%) agreed or strongly agreed that they preferred digital questionnaires over paper questionnaires. All six physicians agreed or strongly agreed that digital pre-visit questionnaires could help to use patient time more meaningfully and increase productivity. Five of six (83.3%) agreed or strongly agreed that they preferred digital over paper forms.

Among intervention participants who used the Idana application, 22 of 22 (100.0%) agreed or strongly agreed that they would use Idana again, 21 of 22 (95.5%) rated it as easy to use, 22 of 22 (100.0%) rated the interface as pleasant, 22 of 22 (100.0%) found errors easy to correct, 22 of 22 (100.0%) rated the questions as clear and 22 of 22 (100.0%) considered the time effort acceptable. Usability ratings from four physicians (2 rheumatologists, 2 orthopedics) were available and favourable. All four available usability evaluations agreed or strongly agreed for future use, ease of use and pleasant design, while three of four (75.0%) agreed or strongly agreed that errors were easy to fix.

### Feedback analysis

Qualitative free-text feedback was available from 40 of 43 patients and from two physicians from the intervention group (rheumatologist and orthopedic). Overall, patient comments were predominantly positive and highlighted digital pre-visit questionnaires as a promising tool to improve consultation preparation and efficiency. The most frequently mentioned perceived benefit was time saving, both for patients and physicians. Patients felt that completing the questionnaire before the visit could help physicians review relevant information in advance, focus the consultation on key issues, reduce repetitive explanations, and potentially shorten waiting times. Several patients also emphasized that answering questions at home allowed more time for reflection, better recall of symptoms and disease history, and less stress or nervousness compared with answering questions during the appointment. Patients also described the digital format as future-oriented, practical, and environmentally beneficial due to reduced paper use. Positive comments regarding the app and questionnaire included easy handling, clear and comprehensive questions, and a concise structure. Some patients suggested that the questionnaires could support better documentation, longitudinal monitoring, and more targeted physician-patient communication. A smaller number of comments raised potential limitations. These included concerns about technical reliability, usability for older patients or those less familiar with digital tools. Some patients also stressed that digital questionnaires should complement, but not replace, direct physician interaction, and that physicians would need sufficient time to review the submitted information before the consultation.

## Discussion

This randomized pilot study evaluated the effect of CAHT combined with a structured, copy-ready summary on documentation completeness for IRD screening. We found that pre-visit CAHT to support screening for inflammatory rheumatic diseases was associated with markedly higher documentation completeness than usual care and well accepted by patients and physicians. Documentation completeness reached 100.0% in the intervention group overall and across all specialty subgroups, whereas routine clinical documentation in the control group varied substantially between specialties. The median control-group score of 46.9% indicates that fewer than half of the predefined screening items were represented in the final note, however it does not establish that the same proportion of clinical information was not elicited during the consultation.

The large difference in documentation completeness is in line with previous studies showing that computer-assisted history-taking can improve the completeness and structure of clinical information [4–6]. This is plausible because the intervention addressed a common workflow vulnerability: relevant symptoms, risk factors, and negative findings may either not be asked about or may be elicited but not systematically documented, particularly when visits are time constrained or findings are not considered immediately relevant [6, 10]. By generating structured narrative summaries before the encounter and allowing simple transfer into the clinical note, CAHT may improve documentation completeness without increasing physician documentation burden and may reduce the cognitive and administrative effort required to record screening-relevant information [7]. The effect appeared most pronounced in orthopedics, where usual-care documentation of inflammatory back pain screening items was particularly low, but was also observed in rheumatology and dermatology. A potential clinical impact is illustrated by one of the five orthopedic intervention patients, who reported all indicative features for axial spondyloarthritis and was referred for rheumatologic evaluation with the structured summary report.

Acceptance and usability findings were also encouraging and are consistent with positive patient experiences reported in early CAHT studies from the 1960s [5] and in recent systematic reviews [6]. Nearly all patients were willing to complete digital pre-visit questionnaires in the future, and acceptance did not differ significantly between patients who had used the system and those who had only received usual care. Among intervention participants, usability ratings were consistently favorable, with all patients indicating that they would use CAHT again and that the questions were clear and the time effort acceptable. Free-text feedback supported these quantitative findings: patients valued the opportunity to answer questions at home, reflect on symptoms, prepare for the consultation, and potentially help physicians focus the visit. At the same time, some comments emphasized that digital questionnaires should complement rather than replace direct physician interaction. These findings are consistent with recent work on the patient self-administered inflammatory arthritis detection tool, in which most patients found the tool easy to complete and both patients and rheumatologists supported referral-stage use when integrated into existing workflows [22].

### Strengths and limitations

Strengths of this study include its randomized design, multicenter recruitment across clinically relevant specialty pathways, blinded assessment of documentation completeness, and inclusion of both quantitative ratings and qualitative feedback. Important limitations include the small sample size and the limited number of physician evaluations. In addition, participants were required to have sufficient German language proficiency and access to an internet-enabled device, which may limit generalizability.

The study did not assess direct clinical outcomes, such as increased diagnosis of inflammatory rheumatic diseases, or workflow outcomes, such as reduced documentation time. Nevertheless, documentation completeness is a relevant intermediate implementation outcome. Without documented screening findings, subsequent clinical action is less likely to occur. In rheumatology, diagnostic accuracy has also been shown to depend on the completeness of available clinical information, irrespective of whether cases are interpreted by large language models [23], medical students [10], or experienced rheumatologists [10, 24]. A further important limitation concerns the relationship between the intervention and the primary outcome. The CAHT-generated summaries and the documentation checklists were based on the same predefined set of specialty-specific items, and the generated summaries were copied largely verbatim into all intervention-group clinical notes. Physician evaluation response rates were limited and should therefore be interpreted with caution. Finally, completion time was not measured systematically, although observed completion times were approximately 5 min in dermatology, 10 min in orthopedics, and 15 min in rheumatology. The ceiling distribution in the intervention arm (median 100.0%, IQR 100.0-100.0) also limits the checklist’s discriminative range and its ability to detect subgroup differences or further improvement. Furthermore, the checklists were not externally validated. Although outcome scoring was blinded, treating physicians were aware of allocation and of the available documentation aid; an observation or Hawthorne effect may therefore have contributed to documentation behavior.

### Future directions

Healthcare burden and time pressure are likely to increase, making task-shifting approaches such as CAHT increasingly important [25]. Future studies should evaluate whether improved documentation translates into earlier recognition of inflammatory rheumatic diseases, more appropriate referrals, shorter diagnostic delays, and better patient outcomes. A larger trial should include predefined implementation outcomes, such as completion rates, time needed by patients and clinicians, physician review behavior, and integration into electronic health records. It should also examine barriers among patients with limited digital literacy, language barriers, or reduced access to digital devices. Future evaluations could also include more granular measures of the accuracy and adequacy of documented content, rather than presence or absence of items alone.

A complementary implementation pathway is to deploy CAHT at the point of referral, allowing structured patient-reported information to accompany referral letters and support specialist triage. This may be particularly useful in health systems with constrained rheumatology capacity and delayed access [26]. Recent work on the patient self-administered inflammatory arthritis detection tool likewise supports referral-stage use when it is integrated into existing workflows [22, 27].

The digital history-taking landscape is rapidly changing with increasingly capable LLM-based clinical chatbots. Recent systems can support dynamic history-taking, multimodal interpretation, and clinical recommendations, i.e. with Google’s multimodal AMIE outperforming primary care physicians on 29 of 32 specialist-rated axes in simulated telehealth consultations [28]. However, LLMs remain probabilistic and may produce hallucinations, inconsistent outputs, or insufficiently validated recommendations. Rule-based CAHT therefore remains valuable for reproducible screening, transparent logic, stable documentation, and structured data capture. Such data could also support research, quality improvement, and future prediction models for risk stratification and referral prioritization. Future systems may combine deterministic questionnaires for core screening with carefully governed conversational AI for clarification, patient support, and clinical reasoning. Broader pragmatic implementation, in collaboration with electronic health record vendors, clinical decision-support developers, health authorities, and payers, could generate valuable real-world evidence.

## Conclusion

Pre-visit computer-assisted history-taking with a structured, copy-ready summary was highly accepted by patients and physicians. Structured patient-reported screening information, when delivered in a directly transferable format, was much more consistently represented in the medical record than under usual care. These findings support further evaluation in larger studies to determine whether this approach improves workflow integration, earlier recognition of inflammatory rheumatic diseases, and clinical outcomes.

## Supplementary Information

Below is the link to the electronic supplementary material.

Supplementary Material 1

Supplementary Material 2

## Data Availability

The raw data and questionnaires will be made available by the authors upon reasonable request.

## Abbreviations

CAHT: Computer-Assisted History Taking
LLM: Large Language Model
