# Supplementary material for: Effect of pre-visit computer-assisted history-taking with a structured copy-ready summary on documentation completeness in screening for inflammatory rheumatic diseases: a randomized pilot study
Source: BMC Health Serv Res. 2026 Oct 9;26:1378. doi: 10.1186/s12913-026-15808-0 (PMC13660116; doi:10.1186/s12913-026-15808-0)
Supplement: Supplementary file 2 [file 12913_2026_15808_MOESM2_ESM.docx]

[Rheumatology questionnaire	2](#_Toc232099962)

[Psoriasis questionnaire	4](#_Toc232099963)

[Back pain questionnaire	5](#_Toc232099964)

[Fig. S1. Rheumatology control group documentation of relevant items	6](#_Toc232099965)

[Fig. S2. Dermatology control group documentation of relevant items	7](#_Toc232099966)

[Fig. S3. Orthopedics control group documentation of relevant items	8](#_Toc232099967)

# Rheumatology questionnaire

1. Do you currently have, or have you ever had, joint or tendon pain or swelling lasting longer than two weeks that was not caused by an injury?

2. Please mark the painful joints.

3. Please mark the sites where you have ever had joint swelling.

4. Have you ever had an entire finger or toe that was swollen and painful without an obvious cause?

5. How long have the joint symptoms been present?

6. How severe was your joint pain during the past week on a scale from 0 to 10?

7. Considering all effects of your disease, how severe was your disease activity during the past seven days?

8. Please assess the course of the pain or swelling.

9. Do you have morning stiffness of the fingers or wrists lasting longer than 30 minutes?

10. Have important activities in your life been affected by bone or joint problems, for example because of difficulties with personal care or the need to change leisure or work activities?

11. Have you already taken medication for the pain?

12. Have you already taken prednisolone or cortisone for the pain?

13. Do you have an underlying rheumatic disease?

14. Are rheumatic diseases known in your family?

15. Do you have any of the following conditions: psoriasis, Crohn's disease, ulcerative colitis, depression, fibromyalgia, diabetes, eye inflammation, osteoarthritis, gout, thrombosis, pulmonary embolism, myocardial infarction, stroke, or another condition?

16. Do you smoke, or have you smoked in the past?

17. Have you used cocaine or injected drugs?

18. What is your weight?

19. What is your height?

20. What is your occupation?

21. Have you had deep-seated low back pain for at least six weeks?

22. Have you had pain or stiffness in both arms or shoulders for at least two weeks when trying to raise your arms above shoulder height?

23. Have you had pain or stiffness in both hips or thighs for at least two weeks when getting up from a chair?

24. Have you had a febrile infection in the past few months?

25. Have you traveled during the past year? If yes, to which countries?

26. Have you taken a new medication during the past three months?

27. Have you ever noticed any of the following: new hair loss, worsening hearing, sores on the fingertips, nailfold bleeding, rheumatoid nodules, increased sensitivity to sunlight with redness after 30 minutes, redness of the cheeks, hardening of the skin, unexplained redness or rashes of the skin, recurrent mouth ulcers, recurrent genital ulcers, or pits or dents in your fingernails or toenails?

28. Have any of the following ever been found in your blood tests: elevated uric acid, rheumatoid factor, elevated inflammatory markers such as C-reactive protein, anti-CCP antibodies, ANA, ANCA, or HLA-B27?

29. Have you had daily, bothersome dryness of the eyes and mouth for more than three months?

30. Have you had recurrent or persistent swollen salivary glands as an adult?

31. Have you noticed symmetrical muscle weakness in your legs or arms?

32. Do you have problems swallowing?

33. In cold conditions, do your fingers or toes suddenly become pale and numb, followed by blue and red discoloration?

34. Have you noticed any of the following symptoms: pain when chewing, headache in the temple area, a painful or thickened blood vessel at the temple, or sudden loss of vision?

35. Do you have shortness of breath?

36. Do you have a dry cough?

37. Do you have lower leg edema?

38. Do you regularly sweat at night so much that you need to change your clothes or bedding?

39. Do you have recurrent episodes of fever above 39 degrees C?

40. Have you unintentionally lost more than 10% of your body weight during the past six months?

41. Have you noticed swollen lymph nodes?

42. During the past two weeks, how often have you been bothered by the following symptoms?

# Psoriasis questionnaire

1. Have you ever had swollen joint(s)?

2. Has a physician ever diagnosed you with arthritis?

3. Do you have pits or dents in your fingernails or toenails?

4. Do you have, or have you ever had, heel pain?

5. Have you ever had an entire finger or toe that was swollen and painful without an obvious cause?

6. Is psoriasis known in your family, for example in your parents or siblings?

7. When you wake up in the morning, do you have morning stiffness in your back lasting about 30 minutes or longer?

8. How severe is your joint stiffness?

9. How would you rate your health?

10. How would you rate your current pain?

# Back pain questionnaire

1. Has your back pain lasted for more than three months?

2. Did your back pain first occur before the age of 45 years?

3. What are the characteristics of your back pain?

4. The back pain started gradually and was not caused by trauma, such as a back injury or strain.

5. When I wake up in the morning, I have morning stiffness in my back lasting about 30 minutes or longer.

6. Movement or exercise improves my back pain.

7. Rest improves my back pain.

8. I sometimes wake up at night because of back pain, especially in the early morning during the second half of the night.

9. I currently have, or previously had, alternating buttock pain, with pain sometimes on the right and sometimes on the left side.

10. I have previously taken a nonsteroidal anti-inflammatory drug for back pain, such as diclofenac or ibuprofen, and the pain improved substantially or resolved completely after taking it.

11. I have, or have had, joint pain with swelling.

12. I have previously been tested for the genetic marker HLA-B27 and the result was positive.

13. I have previously had elevated inflammatory markers, such as C-reactive protein or erythrocyte sedimentation rate, without another condition such as infection explaining this.

14. I have psoriasis.

15. I have inflammatory bowel disease, such as Crohn's disease or ulcerative colitis.

16. I have previously had inflammation of the iris, such as uveitis, iritis, or iridocyclitis.

17. One or more of my first-degree relatives have, or had, ankylosing spondylitis, psoriasis, or inflammatory bowel disease, such as Crohn's disease or ulcerative colitis.

18. Have you ever had an entire finger or toe that was swollen and painful without an obvious cause?

19. I have, or have had, inflammation at tendon insertion sites, for example at the heels or elbows.

20. Axial spondyloarthritis, formerly known as ankylosing spondylitis, is known in my family, for example in my parents or siblings.

Fig. S1. Rheumatology control group documentation of relevant items*.* Heatmap showing whether predefined rheumatology screening items were documented in routine clinical notes among control-group participants. Each column represents one participant, each row represents one screening item. Green cells indicate that the item was documented as present or assessed; red cells indicate that the item was absent from the documentation. Percentages on the right show item-wise documentation completeness across participants, and percentages at the bottom show participant-wise documentation completeness across all items.

Fig. S2. Dermatology control group documentation of relevant items*.* Heatmap showing whether predefined dermatology screening items were documented in routine clinical notes among control-group participants. Each column represents one participant, each row represents one screening item. Green cells indicate that the item was documented as present or assessed; red cells indicate that the item was absent from the documentation. Percentages on the right show item-wise documentation completeness across participants, and percentages at the bottom show participant-wise documentation completeness across all items.

Fig. S3. Orthopedics control group documentation of relevant items*.* Heatmap showing whether predefined orthopedics screening items were documented in routine clinical notes among control-group participants. Each column represents one participant, each row represents one screening item. Green cells indicate that the item was documented as present or assessed; red cells indicate that the item was absent from the documentation. Percentages on the right show item-wise documentation completeness across participants, and percentages at the bottom show participant-wise documentation completeness across all items.
